# Length of stay in the pediatrics emergency department and associated factors among pediatrics patients in Eastern Ethiopia public hospital, Ethiopia 2022

**DOI:** 10.1371/journal.pone.0313146

**Published:** 2025-01-07

**Authors:** Netsanet Melkamu, Amelmasin Faris, Muluken Yigezu, Mickiale Hailu, Anteneh Atle, Manaye Kasahun, Mohammed Kebede, Tsegasew Embiale, Tsinukal Tesfay, Sewmehone Amsalu, Yakob Tadese, Bruck Tesfaye, Gebrehiwot Berie

**Affiliations:** 1 Department of of Nursing, College of Medicine and Health Sciences, Dire Dawa University, Dire Dawa, Ethiopia; 2 Department of Anesthesia, College of Medicine and Health Sciences, Dire Dawa University, Dire Dawa, Ethiopia; 3 Department of Public Health, College of Medicine and Health Sciences, Dire Dawa University, Dire Dawa, Ethiopia; 4 Department of Midwifery, College of Medicine and Health Sciences, Dire Dawa University, Dire Dawa, Ethiopia; 5 Department of of Pediatrics and Child Health Nursing, School of Nursing, College of Medicine and Health Sciences, Wolaita Sodo University, Wolaita Sodo, Ethiopia; 6 Department of Pediatrics and Neonatal Nursing, School of Nursing and Midwifery, institute of Health Sciences, Wollega University, Nekemte, Ethiopia; 7 Department of Pediatrics and Child Health Nursing, College of Health Sciences, Debre Tabor University, Debre Tabor, Ethiopia; Universitair Kinderziekenhuis Koningin Fabiola: Hopital Universitaire des Enfants Reine Fabiola, BELGIUM

## Abstract

**Introduction:**

Patient length of stay is a crucial measure of the emergency department, and it is a vital indicator of health services to evaluate its efficacy, patient care, organizational management, and health care system. Despite this, there are a few studies conducted on pediatric emergency length of stay in developing countries. Therefore, this study serves as input for evidence of pediatric emergency length of stay and associated factors in public hospitals.

**Methods:**

An institution-based cross-sectional study was conducted among children who attended in pediatric emergency department of Eastern Ethiopia public hospital from May 01 to Jun 31, 2022. A total of 761 children were selected by systematic sampling technique and interview using structured questionnaires. After data is collected and cleaned, entered using Epi data version 4.6 and then exported to Stata version 14.1 for analysis. Finally, an AOR with a 95% CI was computed, and variables with a P-value < 0.05 in the multivariable analysis were taken as significant factors for prolonged length of stay.

**Result:**

The prevalence of prolonged length of stay in the emergency ward was 214 (72%). Living in a rural residence ([AOR = 1.65, 95% CI (1.10–2.48)], having a duration of pain > = 12 ([AOR = 1.92, 95% CI (1.13–3.25)], waiting time > = 5 minute ([AOR = 2.24, 95% CI (1.1–4.248541)], having comorbid illness ([AOR = 1.92, 95%CI, 1.13–3.25)], and higher acuity level and absence of medication in the hospital were ([AOR = 2.26, 95%CI (1.02–2.46]) were significantly associated factors for prolonged length of stay.

**Conclusion and recommendation:**

This study revealed that more than two-thirds of children admitted to pediatric emergency had prolonged lengths of stay. This result indicated that higher proportion of the length of stay in pediatric emergency in Eastern Ethiopian public hospitals compare to national. Hence, it is better to give priority to strengthening the focused evaluation of important variables and manage accordingly.

## Introduction

Pediatrics emergency Length of stay (LOS):- is defined as the period starting from registration of patients to their discharge, admission, or referral [1, 2]. Globally, over the last 20 years, Emergency Department (ED) visits have increased by 65% within 9years, and evidence has shown that annual visits have increased faster than the population growth [3, 4]. The daily ED patient flow comprises one-quarter-of of pediatric consultations in Western countries [5]. A study conducted in the United States of America (USA) in 2018 states the annual Pediatric ED visits were nearly 30 million, with 25.6% o being children less than 15 years old. Additionally, study conducted in Australia showed a 21% increase within 2years [6, 7]. This trend may be even more pronounced in developing countries, where a quadruple burden of disease and inadequate resources are prevalent [8].

The ED in the hospital is designed for prompt assessment and stabilization of patients arriving at the hospital in need of immediate care [9]. Upon arrival at the hospital, children are triaged, treated, and then either transferred to a pediatric inpatient ward or discharged. Prolonged ED stay is a worldwide problem that may affect access to healthcare and the quality of service provided, as time is considered a critical component in the quality of ED [10, 11]. However, the cut-offs defining prolonged ED LOS vary across countries ranging between 4 and 48 hours [12], with most Western nations having a range of 4 to 8 hours, most of African having 24–48 hours, and Ethiopia having more than 24 hours [1, 8, 13, 14].

Despite the discrepancy in the cut-off point to determine prolonged LOS, it is a vital factor for ED overcrowding, decreased patient satisfaction, ambulance diversion, and poor clinical outcomes [15]. Crowding and limited resources due to prolonged LOS may reduce access for new admissions, delay care and workups, prolong pain and suffering, as well as delay therapeutic interventions. This puts patients at risk for morbidity, and mortality as well as decreased patient satisfaction. It also increases the socioeconomic burden on parents and limits the proper utilization of healthcare facilities [16, 17]. Unnecessary emergency stays result in overcrowding and overcapacity of ED [17], which is linked to increased patient morbidity, mortality, and dissatisfaction [18, 19]. Children are particulary vulnerable to hazards due to their fragility and differences in developmental stages, so the longer they stay in the ED, the greater the risk. Therefore, identifying potential variables for LOS will aid in developing a tailored intervention to avoid prolonged LOS [2, 20].

Previously, studies have indicated that various factors were associated with prolonged LOS in pediatric emergencies such as arrival time, change in night shift, orders of imaging, referral type, orange triage category, duration of pain, age, and sex [20–22].

While governmental and non-governmental organizations emphasize reducing morbidity and mortality of children, the problems are often higher on this age group [23]. Particularly in low and middle-income countries (LMICs) where late presentation, delayed interventions, financial constraints, unavailability of life-saving equipment and inadequate support services, and insufficient health workforce are prevalent [16]. The situation is worse in Ethiopia, where the ratio of pediatricians per population is low, and there are no emergency pediatrician [24]. To reduce LOS in ED the Ethiopian government aggressively working to reduce child mortality through implementation of different programs, such as standardized Emergency Triage Assessment and Treatment (ETAT) training, triage category, increasing healthcare workforce, and pediatrics emergency unit care [20]. Previous studies have suggested that prolonged LOS can be reduced through triage standards adjustment and ED process redesigns [2]. Nevertheless, pediatric LOS in the ED remains a major problem.

Despite a considerable body of literature from developed countries, the existing information is inadequate in LMICs to inform clinical or service-planning decisions. A deeper understanding of the contributing factors of pediatric ED LOS could help identify patients who require special intervention or resources to prevent a prolonged LOS [20]. Identifying the cause is also essential for developing appropriate interventions and strategies [25]. Moreover, studying LOS is used for resource consumption, effective planning, and management [18, 19]. As children are prone to emergency conditions, studies regarding LOS in ED play a vital role. Despite this, studies related to LOS in pediatric emergencies in Ethiopia are scarce [20]. Therefore, this study assess the Length of Stay in the Pediatrics Emergency Department and associated factors among pediatric patients in Eastern Ethiopia public hospitals, Ethiopia 2022.

## Materials and methods

### Study design and setting

An institutional-based multi-center cross-sectional study was used to assess the length of stay in pediatrics emergency and associated factors among children admitted at Eastern Ethiopia Public Hospital. The study was conducted from May 01 ‐ June 30, 2022, among children admitted at selected Eastern Ethiopia Public Hospitals. Three hospitals were randomly selected considering the availability of the pediatrics emergency unit that includes Chiro General Hospital, Hiwot Fana Referral Hospital and Dilchora Referral Hospital.

Chiro Hospital is a general hospital that is found in the western Hararghe zone, Oromia regional state, Eastern Ethiopia. Chiro Hospital was established in 1969 and Chiro town is located 326 km from Addis Ababa in the eastern direction. According to the 2017 G.C. town health office plan, the estimated total population of the town is 1,607,922. Chiro General Hospital is a zonal hospital that provides emergency management of pediatrics and neonatal care services in the western Hararghe zone. The hospital has given services to more than 1.6 million individuals around the area [26]. The pediatric emergency of Chiro General Hospital contains 14 beds.

Hiwot Fana Specialized Referral Hospital is located in Harar town in the Eastern part of Ethiopia at a distance of 526km away from Addis Ababa, the capital city of Ethiopia. According to the 2007 census report of the Central Statistical Agency, the total population of the region is estimated to be 183, 415, of which 92,316 are males and 91, 099 females. Hiwot Fana Specialized Referral Hospital is the only tertiary hospital that provides different services for approximately 5.8 million people in the catchment area. In the town, there are 45 health facilities (34 health posts, 8 health centers, and 5 hospitals [27].

The third site is Dilchora referral Hospital located in Dire Dawa city administration at a distance of 515 km away from the capital city of Ethiopia, Addis Ababa. Based on the 2019/2020 population projection Central Statistical Agency of Ethiopia: Dire Dawa city administration has a total population of 506,609 (248,238 male and 258,371 females). The administration has 6 hospitals (2 governmental, 2 private, 2 other non-governmental, and 1 other governmental hospital), 15 Health centers (8 urban and 7 rural), 34 health posts, 56 primary, medium, and specialty clinics, and also 30 pharmacies, 42 drug vendor and 4 private hospital pharmacies found in the Administration. Dilchora Hospital is the only referral hospital in the city.

### Population and sample

The study population includes all children who attended in pediatric emergency department at the selected public hospital in Eastern Ethiopia, in 2022. Children attending the pediatric emergency department during the data collection period were included in the study and patients who leave the the wards without notifying the staff and immediate death were excluded.

### Sample size determination and sampling procedure

The sample size was calculated using single population proportion formula based on the following assumptions Za/2:-value of standard normal distribution (z = 1.96) with a confidence interval of 95%, P the prevalence of pediatrics ED LOS, 79.70% [20], d margin of error to be tolerated and taken 3%., and considering 10% non-response rate, final sample size was 761. Finally, data was collected from a total of 761 children attending a pediatric emergency.

**Fig 1:**
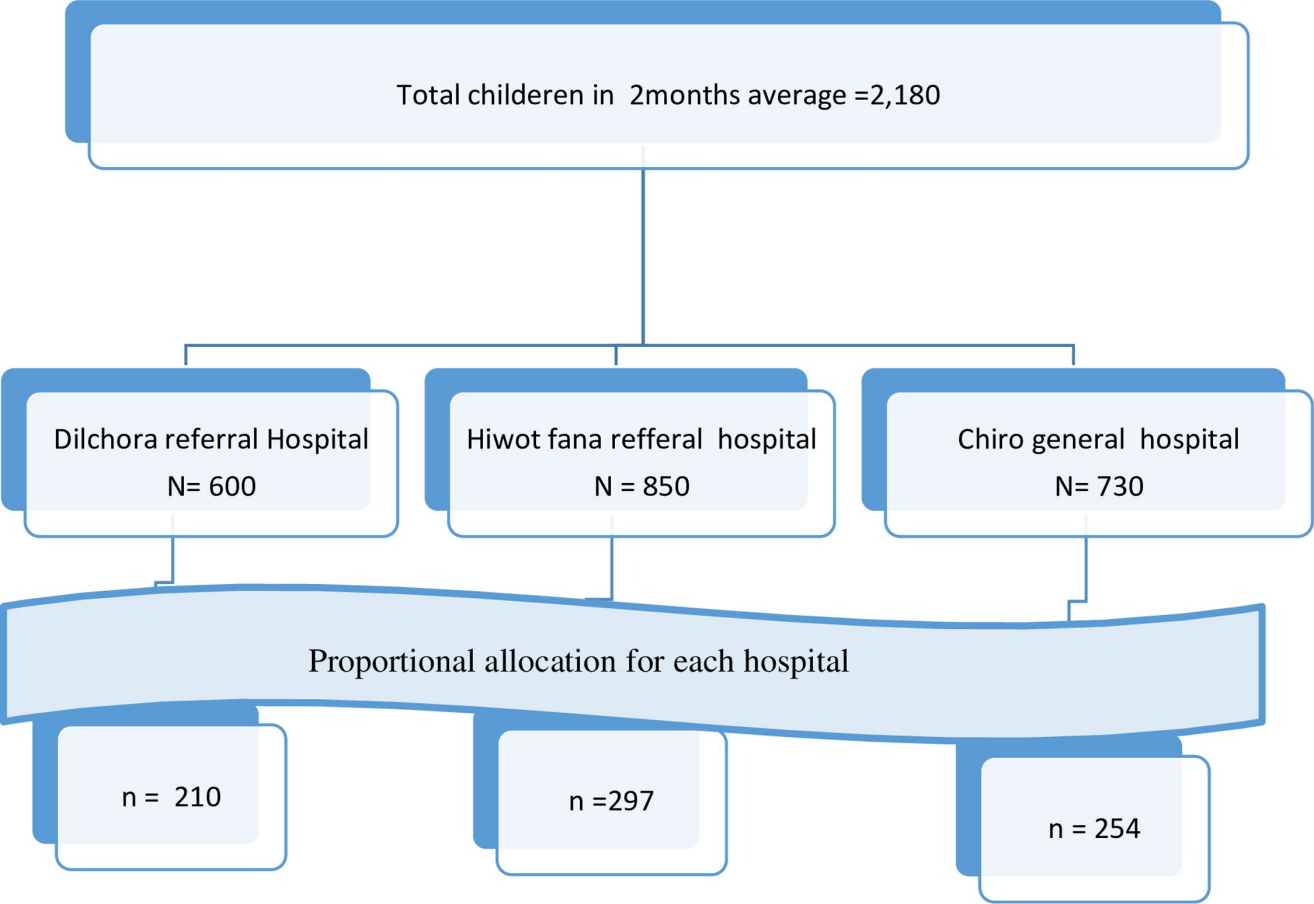
Sampling procedure to select study participants in East Ethiopia public hospitals, Ethiopia from 2022.

Considering the availability of a pediatrics emergency ward three public hospitals in Eastern Hospital were selected. The sampling frame of patients in each of the hospitals was obtained from the previous four months and averages were taken. The total sample size was proportionally allocated for each three hospitals depending on the load of patients registered in the past four months. A systematic random sampling technique was used to select the study participants. To determine the starting point, the lottery method was used. Subsequently, the sampling interval (k) was calculated by dividing the expected number of children visiting the ED during the study period (N) with the determined sample size (n) of respondents (2180/761 = 2.8). Finally, using a systematic random sampling technique, one in every 3 attendant children pairs was selected until the required sample was reached (Fig 1).

### Data collection tools and technique

Data were collected using structured interview questionnaires and standardized tools adapted from a study conducted in Wolaita [20] which was validated. It was prepared in English, then translated into Amharic, Afaan Somalia, Harari, and Afaan Oromo language, and then retranslated to English to check for consistency. The study participants’ language fluency was asked and interviewed based on their choice. A total of thirteen BSc nurses and 3 supervisors were recruited for data collection and supervision. Data collectors were trained for two days before data collection. Finally, the trained data collectors collected data from the patients following presentation, admission, and before discharge from the ED through respondent interviews, observation, and medical record review.

The data collection procedure will be illustrated as follows: first, eligible patients will be identified by data collectors at the triage room while resident staff triage patients on arrival. Hence at arrival, time and other presentation characteristics were recorded in the triage room. Following this, other information such as socio-demographic characteristics and other organizational-related factors was obtained through interviews, chart review, and observation of patients at different treatment points. Finally, information such as diagnostic investigations and overall treatments were recorded from medical records, whereas the overall length of stay at the department and final disposition were recorded right before the patient was discharged from the ED. Later, essential time-related factors and the overall length of stay between presentation and discharge from ED.

### Variables of the study

The dependent variable was Length of stay (prolonged, not prolonged), whereas the independent variables were socio-demographic (age, sex, residence, language, income, religion, mode of arrival, education level, marital status, and occupation), time-related factors (time of arriving, date of arriving, duration of pain, and waiting time), clinically related factors (chief compliant, referral status, triage category, prior treatment, diagnosis, comorbid illness, mental status and outcome of the patient), organizational related factors (examiners, diagnostic investigations, discharge, admission, refer, shift change and experience)

### Operational definition

**Pediatrics emergency department length of stay:** is the total length of time for all emergency case attendants who stay in the emergency room from minutes to hours (days) starting from registration. According to Ethiopian hospital services transformation guidelines: patients’ LOS in the emergency department should not be greater than 24 hours. Then transfer to wards has been facilitated before 24 h for proper inpatient admission if necessary [13]. Waiting time is calculated by subtracting the ED registration time from the time a patient was placed in an ED treatment room [21]. Prolonged length of stay: defined as a patient stays in the emergency department > 24 hours [13, 20]. Not prolonged length of stay: when a patient stays in the emergency department < 24 hrs. [13, 20].

### Ethical approval and consent to participate

Ethical clearance was obtained from the Dire Dawa University Institutional Review Board in a letter protocol number DDU-IRB-2022-070 and a Permission letter was obtained from Hiwot Fana referral hospital, Chiro General Hospital, Dilchora referral hospital management, and the pediatric emergency unit head. The study participants were informed about the purpose and importance of the study. The participants were assured the right to refuse or withdraw at any time. Finally, voluntary written and signed informed consent was obtained from the study participant or their parents/legal guardians before data collection. The Confidentiality of the study participants was maintained throughout the research process by giving the code for participants. The study participants were also informed that data were kept private and confidential, and only used for research purposes.

### Data quality control and management

To ensure quality of data tool translated into the local languages Amharic, Afaan Somalia, Harari, and Afaan Oromo. The data collectors were recruited based on their language fluency in Amharic, Afaan Somalia, Harari, and Afaan Oromo. The recruited data collectors trained for two days on the objective, confidentiality of information, informed consent, and techniques of interviews. Three supervisors supervised the data collectors and reported daily. Before data collection, pretest was conducted on 5% of the total sample and modified accordingly. The collected data checked for completeness and consistency on the day of data collection. Simple frequencies and cross-tabulation were done for missing values and cross-checked with hard copies of the collected data.

### Data analysis

Data clean up and cross-checked before analysis and entered to Epi data version 4.6, finall exported to Stata version 14.1 for analysis. The descriptive statistics computed to summarize the descriptive results and presented in texts, tables, and figures. The model fittnes checked by the Hosmer-Lemeshow goodness of a fit test and ROC curve (Receiver Operating characteristic Curve). Multicolinarity checked using VIF(Variance Inflation Factors). A bi-variable logistic regression model was used to assess statistical association between the outcome variable and every independent variable. Variables that showed statistical significance during bi-variable analysis at P-value ≤ 0.25 entered to multivariable logistic regression. Finally the strength of association are taken with adjusted odds ratios (AOR) with a 95% Confidence interval at a p-value < 0.05.

## Result

### Socio-demographic characteristics

In this study, a total of 761 children interviewed and had response rate of 100%. About 400(52%) of study participants were female. The median age of study participants was 2years and had IQR of 5.3. About 358 (47%) of respondents are Muslim religions followers, followed by Orthodox religion 216 (28%). Concerning the educational status of the caregivers, more than 2/3rd of study participants have formal education and can speak more than one languaghe (Table 1).

**Table 1:** Socio-demographic characteristics of respondent at the pediatric emergency department in Eastern Ethiopia public hospitals, Ethiopia, 2022(N = 761).

Variables	Category	Frequency	Percent
Age of the children	<1 years	323	42.44
	1–5 years	238	31.28
	>5 years	200	26.28
Sex of the children	Male	361	47.44
	Female	400	52.56
Residence	Urban	369	48.49
	Rural	392	51.51
Religion	Orthodox	216	28.38
	Muslim	358	47.04
	Protestant	114	14.98
	Catholic	52	6.83
	Others	21	2.76
Ethnicity	Amhara	124	16.29
	Oromo	431	56.64
	Somali	77	10.12
	Harari	93	12.22
	Others	36	4.73
Marital status of the caregivers	Married	540	70.96
	Never married	121	15.90
	Living together	57	7.49
	Others	43	5.65
Educational status of the caregivers	No formal education	204	26.81
	Primary education	210	27.60
	High school	141	18.53
	Secondary School	115	15.11
	Above Secondary school	91	11.96
Language spoken	One	199	26.15
	Two	342	44.94
	More than two	220	28.91
Monthly Income	< = 500	34	4.47
	500–1000	48	6.31
	1001–2000	49	6.44
	>2000	630	82.78
Distance to hospital in km	<7	260	34.17
	7–25	136	17.87
	>25	365	47.96
Occupation	Governmental	176	23.13
	Housewife	50	6.57
	Private	209	27.46
	Farmer	176	23.13
	Daily laborer	133	17.48
	Others	17	2.23

### Presentation time characteristics

Regarding presenting characteristics, half of the study participants presented during the morning, and about 548(72.01%) of the study participants arrived on the weekdays. About 316 (41.52%) participants used taxis as a means of transportation, followed by an ambulance (Table 2).

**Table 2:** Presentations time characteristics of respondent at the pediatric emergency department in Eastern Ethiopia public hospitals, Ethiopia, 2022(N = 761).

Variables	Category	Frequency	Percent
Time of arrival	Morning	377	49.54
	Afternoon	250	32.85
	Night	134	17.61
Day of |arrival	Weekdays	548	72.01
	Weekends	193	25.36
	Holydays	20	2.63
Mode of arrival	By taxi	316	41.52
	By ambulance	64	8.41
	By public transport	311	40.87
	By foot	33	4.34
	Others	37	4.86

### Clinical and organizational related factors

**Fig 2:**
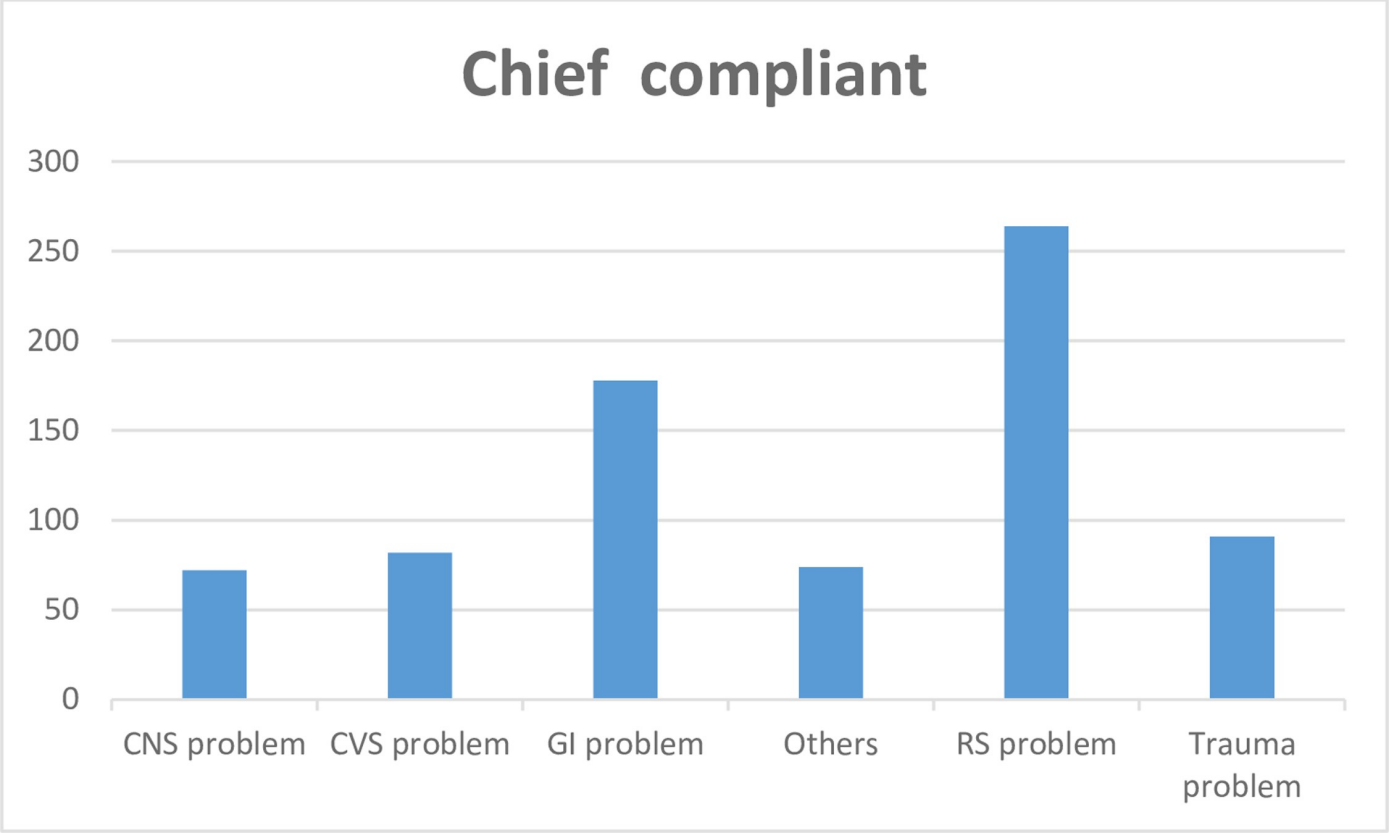
Chief complaints of the respondents during presentations at the pediatric emergency department in Eastern Ethiopia public hospitals, Ethiopia, 2022(N = 761).

As shown below (Fig 2), the leading chief complaints of study participants in pediatrics emergency on admission were respiratory-related complaints 264 (34.69%) followed by Gastroenteritis infection 178(23.39%).

As shown below (Table 3), the clinical characteristics of study participants during admission, more than half of study participants went directly to the hospital 401(52.69%), and the majority of them had taken various medications 573(75.30%) before arrival to the hospital. Concerning preexisting comorbid disease, the majority of respondents, 596 (78.32%), had not had the comorbid disease, and nearly 45% of study participants waited more than 5 minutes. Regarding the triage category, the majority of them are categorized under red level 222(29.17), followed by yellow category 216(28.38). Regarding laboratory investigation, almost all study participants had an ordered investigation. Nearly two-thirds of the study participants had sent a laboratory investigation. In addition, most study participants had gotten order medication in the hospital 681 (91.90%).

**Table 3:** Clinical and organizational related factors at the pediatric emergency department in Eastern Ethiopia public hospitals, Ethiopia, 2022(N = 761).

Variables	Category	Frequency	Percent
Referral status	Self-referral	401	52.69
	From health center	214	28.12
	Others	146	19.19
Preexisting comorbidity	Yes	165	21.68
	No	596	78.32
prior treatment	Yes	573	75.30
	No	188	24.70
Triage category	Red	222	29.17
	Orange	200	26.28
	Yellow	216	28.39
	Green	123	16.16
Waiting time	≤5 minutes	421	55.32
	>5 minutes	340	44.68
Got ordered investigations in the hospital	Yes	681	89.49
	No	80	10.51
Got ordered medication in the hospial	Yes	501	65.83
	No	260	34.17
Staff shift exchange experience	Yes	648	85.15
	No	113	14.85

### Pediatrics emergency department length of stay

**Fig 3:**
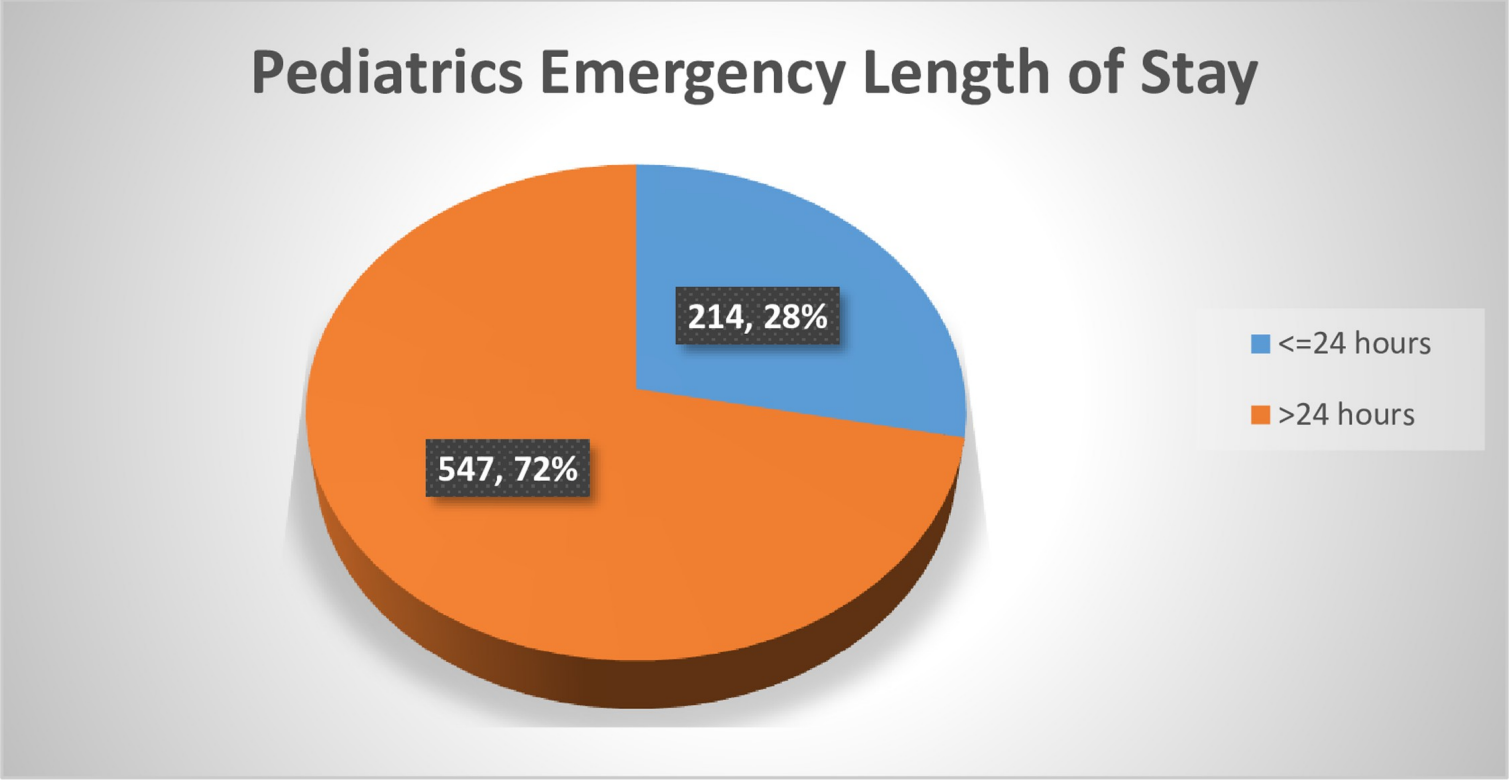
Length of Stay in the pediatric emergency department in Eastern Ethiopia public hospitals, Ethiopia, 2022(N = 761).

The prevalence of pediatrics ED LOS greater than 24 hours was 547(72%). The children stayed in the ED for a minimum of 1.30 hours. and a maximum of 362 with a median of 48 hours (Fig 3).

### Bi-variable and multivariable logistic regression analysis analysis

**Fig 4:**
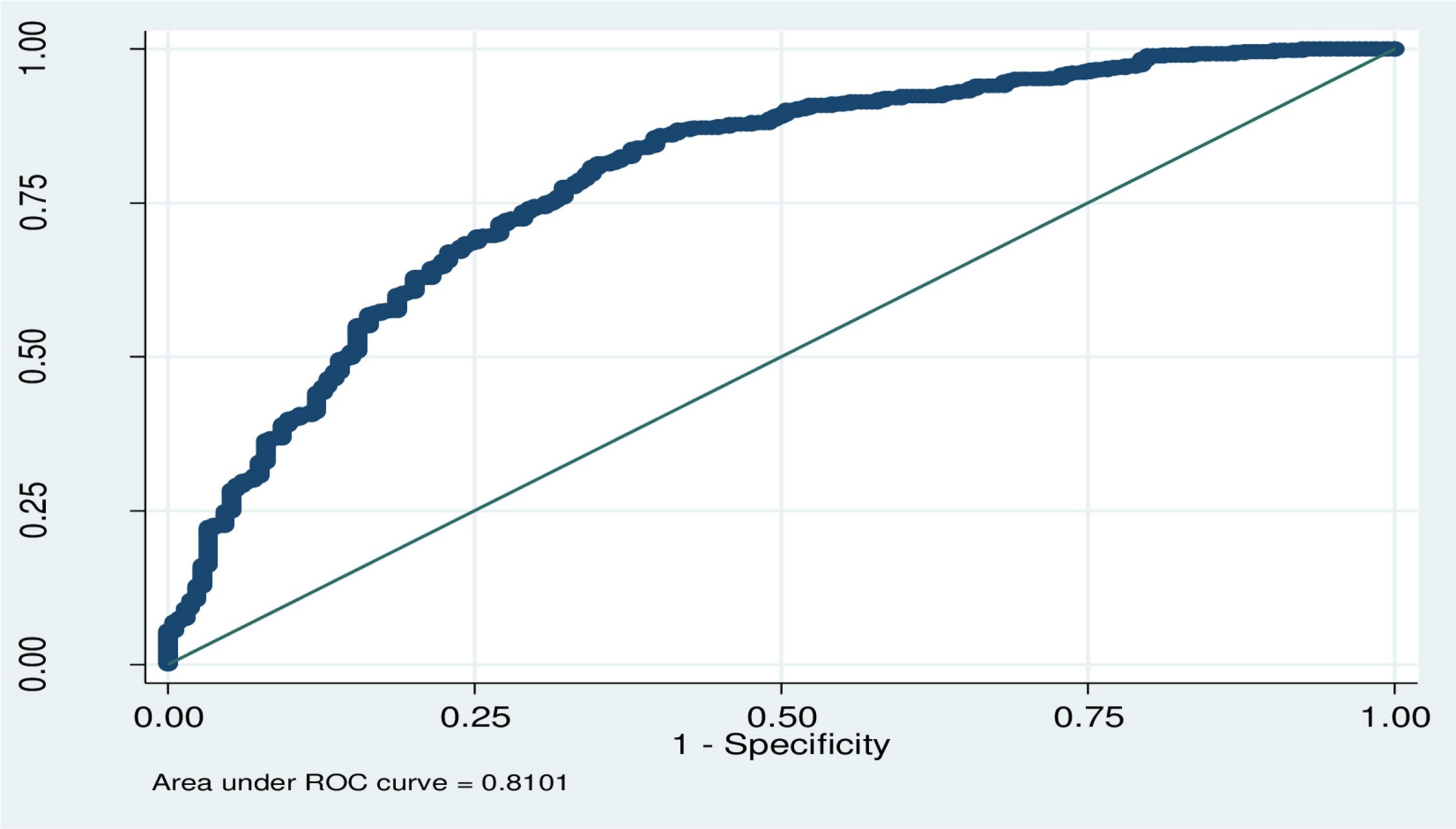
Shows model analysis to assess length of Stay in the pediatric emergency department and associated factors in Eastern Ethiopia public hospitals, Ethiopia, 2022(N = 761).

In the Bi-variable logistic regression age of the children, sex, educational level, residence language, duration of pain, distance from the hospital, time arrivals, waiting time, mode of arrival, refer status, comorbidity, prior treatment, triage category and medication whereas, on the multivariable logistic regression variables such as residence, duration of pain, waiting time, comorbidity, triage category, and presence of medication were significantly associated with the prolonged length of stay (Table 4). The appropriateness of the model was checked using the Hosmer-Lemeshow test (P = 0.51) and graphically ROC = 0.81 (Fig 4).

**Table 4:** Bi-variable and multivariable analysis to assess length of stay in the pediatric emergency department and associated factors in Eastern Ethiopia public hospitals, Ethiopia, 2022(N = 761).

Variables	Category	Length of stay In hours		COR	AOR	P>Z	[95% CI]	
		≤24 hrs	>24 hrs					
Age	<1 years	90	209	1	1			
	1–5 years	84	202	0.78	1.12	0.59	0.74	1.70
	>5 years	40	136	1.61	1.93	0.00	1.18	3.15
**Sex**	Male	110	251					
	Female	104	296	1.25	1.34	0.12	0.92	1.95
**Educational level**	No formal education	50	154	1	1			
	Primary	50	160	1.03	1.35	0.26	0.80	2.31
	High school	55	86	0.50	0.71	0.22	0.42	1.23
	Secondary	27	88	1.05	1.30	0.39	0.70	2.42
	Above secondary	32	59	0.59	0.62	0.20	0.35	1.25
**Residence**	Urban	131	238					
	Rural	83	309	2.05	1.60	**0.02**	1.07	2.39
**Language**	One	56	143	1	1			
	Two	96	246	1.03	0.65	0.07	0.41	1.04
	Three	51	130	0.99	0.98	0.95	0.58	1.67
	More than three	11	28	0.99	1.12	0.78	0.47	2.72
**Time of arrivals**	Morning	125	252	1	1			
	Afternoon	62	188	1.50	1.13	0.54	0.79	1.86
	Night	27	107	1.96	1.57	0.10	0.90	2.74
**Mode of arrivals**	Foot	12	21	1	1			
	Taxi	104	21	1.16	1.62	0.28	0.67	3.97
	Ambulance	18	46	1.46	1.16	0.77	0.40	3.40
	Public transport	66	245	2.12	1.50	0.38	0.59	3.83
	Others	14	23	0.93	1.09	0.87	0.36	3.33
**Refer status**	Self-referral	148	253	1	1			
	Health center	44	170	2.26	1.56	0.08	0.95	2.58
	Others	22	124	3.29	1.51	0.16	0.85	2.70
**Comorbidity**	No	188	408	1	1			
	Yes	26	139	2.46	1.95	**0.01**	1.16	3.29
**Prior treatment**	Yes	149	424					
	No	65	123	1.50	1.20	0.41	0.77	1.87
**Triage category**	Green	58	65	1	1			
	Red	47	175	3.32	2.59	**0.00**	1.46	4.61
	Orange	46	154	2.98	2.34	0.00	1.33	4.16
	Yellow	63	153	2.16	2.48	0.00	1.46	4.23
**Medication**	Yes	167	334	1	1			
	No	47	213	2.26	1.61	**0.03**	1.05	2.49
**Distance from the hospital**	<7	103	157	1	1			
	7–25	34	102	1.96	1.67	0.07	0.95	2.95
	>25	77	288	2.45	1.19	0.49	0.72	1.99
**Waiting time**	≤5 minute	161	260	1	1			
	>5 minute	53	287	3.35	2.93	**0.00**	1.97	4.36
**Duration of pain**	≤12 hours	55	85	1	1			
	13–24 hours	44	57	0.83	0.60	0.11	0.33	1.12
	25–48 hours	32	88	1.77	1.28	0.43	0.69	2.38
	49–72 hours	43	111	1.67	1.32	0.36	0.72	2.44
	≥73 hours	40	206	3.33	2.02	**0.01**	1.13	3.64

This study showed that the odds of prolonged LOS among children age > 5 years are 2 times than the lower age group ([AOR = 2, 95% CI (1.18 3.14)]. Keeping other variables constant, children residing in rural areas were 1.65 times ([AOR = 1.65, 95% CI (1.10–2.49)] more likely to prolong than urban. Those who have comorbid illness had 1.92 times the odds of prolonged emergency than their counterpart ([AOR = 2, 95% CI (1.14–3.26)]. Regarding the triage category, children in the red, orange, and yellow had odds of 2.9, 2.5, and 2.7 times more likely to prolong in the emergency than the green category, respectively. Controlling other variable constants, children who got medication in the hospital were 37.2% less likely to prolong than those who didn’t get it in the hospital. Concerning waiting time, children who waited without treatment for more than 5 minutes had 2.24 times the odds of prolonged stay compared to their counterparts. Keeping other variables constant odds of children having pain for more than 3 days had 3.91 times more to prolonged than less than 12 hours.

## Discussion

Most patients view the ED as their front doors to the healthcare system; their time in the ED, doesn’t end until they are either sent home, admitted to the hospital, or transferred to another facility. Length of stay in the ED is one of the most important metrics for evaluating an ED’s overall effectiveness and level of care. The duration of stay in an ED varies depending on the country and different factors. Therefore, this study assesses the LOS in the pediatric emergency department and associated factors among pediatric patients in Eastern Ethiopia public hospitals, Ethiopia 2022.

The overall proportion of the prolonged LOS in the pediatrics emergency unit was 72% [95%, CI (68.-75)]. This result is in line with a study conducted in Botswana 72.5% [28]. However, it’s higher than the studies conducted in Egypt, Nigeria, Netherlands, Iran, and Indonesia [11, 16, 21, 29]. These differences may be due to the differences in organizational structure, administration, the health system of the organization, and the availability of organized services [17]. In addition factors such as cut of point to define prolonged LOS, difference in inclusion criteria and study year may play significant roles. Studies conducted in Egypt and Iran used the mean and accelerated failure time (AFT) model as a cut-off point to define prolonged LOS. Additionally, studies conducted Netherlands and Iran use cut-off points of 4 hrs. and 6 hrs. respectively. Furthermore, studies show that variations in staffing, infrastructure, accessibility of medical equipment, and other organizational processes may contribute to this variation. Moreover, in a study conducted in Nigeria, the majority of study participants were reviewed within 15 minutes, had timely consultation and adequate subspecialties may contributed to the discrepancy [8, 16, 21].

However, length of stay in our studies area were lower than studies conducted Wolaita, 79.70% and southern Ethiopia, 91.5% [17, 20]. This difference may be due to the difference in study year conducted i.e. during COVID 19 pandemic and both studies conducted in single institutions, additionally study conducted in southern Ethiopia includes adult age and have stated lack of admission of beds to inpatient as a factor for prolonged LOS [17, 30].

Concerning factors associated, the study showed that the odds of prolonged LOS among children aged >5 years were 1.93 times odds of prolonged than lower age group ([AOR = 1.93, 95% CI (1.18–3.15)]. This result is in line with studies conducted in Iran, Botswana, and Taiwan [22, 28, 30]. This may be due to increasing age associated with increasing risk of having comorbidity and disease complexity [31].

Regarding geographical residents, the odds of prolonged length of stay among children residing in rural were 1.65 times [(AOR = 1.6, 95% CI (1.07 2.39)] more likely to prolong than in urban. This is supported by a systematic review conducted in England which concludes that geographical variation affects LOS [32]. In addition, studies conducted in China, Italian and, Nigeria have reported that place of residence has a significant relationship with pediatric emergency LOS [2, 16, 30, 33, 34]. Furthermore, a cross-sectional study done in south Iran revealed that LOS has a significant relationship with place of residence [14]. This may be due to children mostly depending upon the caregivers, so families from rural residences will have delays in the decision due to lack of health care service, indirect cost, lack of transport, and other reasons [2, 35].

Another factor for prolonged ED LOS in our study was having comorbid illness, those who have comorbid illness had 1.92 times odd of prolonged in emergency than their counterpart ([AOR = 1.95, 95% CI (1.16 3.29)]. This result is in line with study Australia and, Netherlands [36, 37]. In addition, a systematic study conducted worldwide has stated that comorbidity was associated with ED admission, PLOS as well as health care costs [38, 39]. This may be due to these patients mostly being admitted to tertiary hospitals with multiple chief complaints that require several diagnostic investigations, consultation, and sophisticated care [21]. Therefore, the complexity of the care and delays caused by diagnostic testing and consultations could contribute to PLOS **[21, 40].**

The present study showed triage levels having red, orange, and yellow categories had nearly 2.5 times the odds of prolonging the length of stay compared to the green category. This result is supported by studies conducted in Wolaita, Switzerland, USA, and Taiwan, in 2017 [2, 11, 20, 41, 42]. This might be due to the patients on triage level II have high acuity with increased disease severity, the patients come in need of lots of care, and the emergency interventions require more time, and intensive treatment. In addition, triage level interpretation, and unique characteristics of the local ED and national health system are different at other hospitals [11, 43]. This might be due to when the acuity level of the disease is increased, the patients come in need of lots of care, and the emergency interventions require more time, intensive treatment is given, likewise, the nature of the disease in the pediatrics population by itself is challenging to manage easily for health professionals, and the higher the acuity level the more challenged and physicians require more time to decide the appropriate place for the patient’s weather inpatient ward, refer to other hospital or discharge. In addition, patients with high acuity levels may need experts (pediatrician) and investigation, this will increase waiting time for the pediatrician and to got ordered investigation, which may contribute for prolonged LOS [22].

Controlling other variable constants those who got medication in the hospital were 39.2% less likely to prolong than those who didn’t have it in the hospital. This is in line with a study conducted in Wolaita, Ethiopia. This may be due to not getting medication in the hospital will make the patients search the drugs outside the hospital compound which hinders the intake of prescribed drugs and adversely affects the patient’s recovery [20].

Concerning waiting time those who have waited without treatment for more than 5 minutes had 2.93 times odd prolonged stay compared to their counterpart. This is comparable with studies conducted in the USA, Botswana, Taiwan, and South Africa [28, 41, 42, 44]. This could be explained by the longer the patients stay in waiting area, its ends up crowding of ED. This would delayed the diagnosis and treatment of their current conditions that would increase the complexity and worsening of the diseases [45]

Keeping other variables constant, the odds of children having pain for more than 72 hours were 2 times prolong their stay compared to those with pain reported for less than 12 hours. This result supported by a study conducted in Wolaita, Ethiopia. This could be explained by patients that had duration of pain of 13–24 hours without treatment might come with an acute life-threatening emergency that complex and worse disease progression [20].

## Conclusion and recommendation

### Conclusion

More than two-thirds of children admitted in pediatric emergencies had prolonged LOS. This result indicates a higher proportion of the LOS in a pediatric emergency in Eastern Ethiopia public hospitals compared to national. Being resides in rural, having a higher duration of pain, time arrivals, long waiting times, having a comorbid illness, having red, yellow, and orange triage categories, and the presence of medication in the hospital were significant factors associated with prolonged LOS.

### Recommendation

Based on this study we would like to recommend the Ethiopian minister of health and other concerned bodies to enhance the accessibility of health services to the rural residents. Designating a temporary department to accommodate admitted patients who are waiting for beds and strengthening the hospital pharmacy to accommodate the needs of customers. To strengthen the monitoring and evaluation of the LOS in ED and use different strategies to decrease the length of stay. The health workers also need to provide health information for parents or guardians regarding the importance of early access of medical service for children health. Further research that will incorporates important variables using a mixed study design and further research using a prospective cohort study design. Parents (caregivers) need to be aware of the pain of their children and promptly visit health facilities. The health workers better consider this variable being in rural residents, duration of pain, time of arrival, waiting time, having a comorbid illness, higher triage level, and absence of medication in the hospital were variables that prolonged LOS. Therefore, health workers need to provide more emphasis on these variables to reduce the LOS in the ED.

## Supporting information

S1 File (DTA)

## Abbreviations

ED: Emergency Department
LMICs: Low and Middle-Income Countries
EM: Emergency Medicine
LOS: Length of Stay
ETAT: Emergency Triage Assessment and Treatment
PLOS: Prolonged Length of Stay
ETB: Ethiopian Birr
USA: United States of America
FMHO: Federal democratic republic of Ethiopia ministry of health
